# Institutional case volume and mortality after aortic and mitral valve replacement: a nationwide study in two Korean cohorts

**DOI:** 10.1186/s13019-022-01945-0

**Published:** 2022-08-20

**Authors:** Karam Nam, Eun Jin Jang, Jun Woo Jo, Jiwon You, Jung-Bin Park, Ho Geol Ryu

**Affiliations:** 1 Department of Anesthesiology and Pain Medicine, Seoul National University Hospital, Seoul National University College of Medicine, 101 Daehak-ro, Jongno-gu, Seoul, 03080 Republic of Korea; 2 Department of Information Statistics, Andong National University, Andong, Gyeongsangbuk-do Republic of Korea; 3 Department of Statistics, Kyungpook National University, Daegu, Republic of Korea

**Keywords:** Aortic valve replacement, Case volume, Mitral valve replacement, Surgical prognosis, Volume-outcome relationship

## Abstract

**Background:**

There are only a handful of published studies regarding the volume-outcome relationship in heart valve surgery. We evaluated the association between institutional case volume and mortality after aortic valve replacement (AVR) and mitral valve replacement (MVR).

**Methods:**

Two separate cohorts of all adults who underwent AVR or MVR, respectively, between 2009 and 2016 were analyzed using a Korean healthcare insurance database. Hospitals performing AVRs were divided into three groups according to the average annual case volume: the low- (< 20 cases/year), medium- (20–70 cases/year), and high-volume centers (> 70 cases/year). Hospitals performing MVRs were also grouped as the low- (< 15 cases/year), medium- (15–40 cases/year), or high-volume centers (> 40 cases/year). In-hospital mortality after AVR or MVR were compared among the groups.

**Results:**

In total, 7875 AVR and 5084 MVR cases were analyzed. In-hospital mortality after AVR was 8.3% (192/2318), 4.0% (84/2102), and 2.6% (90/3455) in the low-, medium-, and high-volume centers, respectively. The adjusted risk was higher in the low- (OR 2.31, 95% CI 1.73–3.09) and medium-volume centers (OR 1.53, 95% CI 1.09–2.15) compared to the high-volume centers. In-hospital mortality after MVR was 9.3% (155/1663), 6.3% (94/1501), and 2.9% (56/1920) in the low-, medium-, and high-volume centers, respectively. Compared to the high-volume centers, the medium- (OR 1.97, 95% CI 1.35–2.88) and low-volume centers (OR 2.29, 95% CI 1.60–3.27) showed higher adjusted risk of in-hospital mortality.

**Conclusions:**

Lower case volume is associated with increased in-hospital mortality after AVR and MVR. The results warrant a comprehensive discussion regarding regionalization/centralization of cardiac valve replacements to optimize patient outcomes.

**Supplementary Information:**

The online version contains supplementary material available at 10.1186/s13019-022-01945-0.

## Background

Since the first demonstration of the inverse relationship between procedural case volume and surgical mortality in 1979 [1], numerous studies have followed seeking the volume-outcome relationship in various surgical procedures [2–4]. The improved outcomes associated with case volume leads to the discussion of regionalization/centralization of high-risk surgical procedures [5].

Cardiac surgery is a procedure that carries one of the highest risk that requires systemized, multidiscipline, and comprehensive perioperative care, as well as sophisticated and skilled surgical technique [6]. Many studies have shown the positive volume-outcome relationship for coronary artery bypass grafting [7–9], but relatively few for heart valve surgeries, including aortic valve replacement (AVR) [10–12] and mitral valve replacement (MVR) [13, 14], the two most commonly performed surgeries excluding coronary artery bypass grafting [15]. Furthermore, the volume-outcome relationship in AVR or MVR has been evaluated largely in the US [11–14]. The aim of this study was to assess the association between institutional case volume and postoperative mortality in patients undergoing AVR and MVR in Korea. Therefore, a nationwide study in the two cohorts were performed using a Korean healthcare insurance database.

## Methods

The present study was a Korean population-based, retrospective observational study, which analyzed patients who underwent AVR and MVR, separately. Data were obtained from the National Health Insurance Service (NHIS) database of Korea. The NHIS is a single payer government warranted health insurance system with more than 97% mandatory coverage of Korean residents [16, 17]. The study protocol was exempt from the review by the Institutional Review Board of Seoul National University Hospital because of its retrospective and anonymous nature (no. 1803-058-928). Written informed consent was also waived by the review board.

### Study population 1: aortic valve replacement

All adult patients (≥ 18 years-old) who underwent AVR with or without concurrent coronary artery bypass grafting from January 2009 to December 2016 in Korea were identified using the NHIS billing code for AVR (O1793). Sutureless and transcatheter AVR were excluded from the analysis. Patients who underwent other heart valve or thoracic aorta surgery at the same time were also excluded.

### Study population 2: mitral valve replacement

All adult patients who underwent MVR with or without concurrent tricuspid valve repair or surgical ablation of atrial fibrillation during the same study period in Korea were included using the NHIS billing code for MVR (O1792). Patients who underwent concurrent surgeries on other heart valves or thoracic aorta were excluded from the analysis.

### Study outcomes, case volume, and risk factors

The primary outcome was in-hospital mortality after AVR and MVR. Secondary outcomes included 1 year mortality and cumulative all-cause mortality.

Institutional case volume of AVR and MVR was defined as the average number of each surgery performed per year during the study period. Redo AVR and MVR (O1796 and O1795, respectively) were also included for the calculation of institutional case volume. AVR centers were categorized into three groups according to the case volume: the low- (< 20 cases/year), medium- (20–70 cases/year), and high-volume centers (> 70 cases/year). MVR centers were also grouped as the low- (< 15 cases/year), medium- (15–40 cases/year), or high-volume centers (> 40 cases/year). These cut-off values were determined based on visual inspection of the scatterplots of institutional case volume.

Relevant risk factors, such as age, sex, preoperative medical history (hypertension, dyslipidemia, diabetes mellitus, extracardiac arteriopathy, chronic lung disease, renal impairment, atrial fibrillation, angina pectoris, myocardial infarction, and congestive heart failure), nature of the surgical procedure (emergent or elective), and the amount of perioperative red blood cell transfusions, were collected from the NHIS database. Preoperative medical history data documented in the NHIS database were retrieved using the International Classifications of Diseases, 10th revision codes. Aortic valve disease status (stenosis, insufficiency, stenoinsufficiency, or unspecified) and concurrent coronary artery bypass grafting were obtained for the AVR population, and rheumatic mitral valve, concurrent atrial fibrillation surgery, and concurrent tricuspid valve repair for the MVR population. Infective endocarditis was collected for both populations.

### Statistical analysis

The patient characteristics were presented as mean ± standard deviation or median [interquartile range (IQR)] for continuous variables, and number (proportion) for categorical variables. The analysis of variance and the chi-squared test were performed to compare continuous and categorical variables, respectively, between the case volume strata.

Logistic regression analysis was used to compare the risk of in-hospital mortality after AVR and MVR, according to institutional case volume. Univariable analyses were performed with all relevant variables that could be extracted from the database. After univariable analyses, all covariates were entered to the multivariable model and adjusted for without applying a variable selection method. In addition, year of surgery was adjusted for as a continuous variable.

The risk of 1 year mortality after AVR and MVR was compared between the case volume strata in the same fashion as in-hospital mortality. The Kaplan–Meier curves of cumulative all-cause mortality were compared among the case volume strata using the log-rank test. Also, Cox proportional hazard model analysis was performed using the same multivariable analysis protocol, but without the year of surgery.

All statistical analyses were performed using R version 4.0.0 (R Foundation for Statistical Computing, Vienna, Austria) and SAS 9.4 (SAS Institute, Cary, NC). A *P* value of < 0.05 was considered to be statistically significant.

## Results

The overall in-hospital mortality rate following AVR was 4.6% (366/7875). In the low-, medium-, and high-volume centers, in-hospital mortality rates were 8.3% (192/2318), 4.0% (84/2102), and 2.6% (90/3455), respectively. The overall in-hospital mortality rate after MVR was 5.9% (305/5084). In-hospital mortality rates were 9.3% (155/1663), 6.3% (94/1501), and 2.9% (56/1920) in the low-, medium-, and high-volume centers, respectively. Patient characteristics for both AVR and MVR study populations are summarized in Table 1.

**Table 1:** Characteristics of the two study cohorts according to the case volume strata

	Aortic valve replacement (n = 7875)				Mitral valve replacement (n = 5084)			
	Low-volume (< 20 cases/year)	Medium-volume (20–70 cases/year)	High-volume (> 70 cases/year)	*P*	Low-volume (< 15 cases/year)	Medium-volume (15–40 cases/year)	High-volume (> 40 cases/year)	*P*
No. of patients	2318	2102	3455		1663	1501	1920	
Age (years)	66 (12)	67 (12)	67 (12)	< 0.001	60 (13)	59 (13)	58 (12)	0.001
Female	939 (40%)	848 (40%)	1446 (42%)	0.442	912 (55%)	899 (60%)	1259 (66%)	< 0.001
Hypertension	1500 (65%)	1388 (66%)	2319 (67%)	0.165	983 (59%)	919 (61%)	1182 (62%)	0.280
Dyslipidaemia	761 (33%)	624 (30%)	1248 (36%)	< 0.001	332 (20%)	249 (17%)	362 (19%)	0.046
Diabetes mellitus	482 (21%)	446 (21%)	647 (19%)	0.041	211 (13%)	188 (13%)	212 (11%)	0.251
Extracardiac arteriopathy	353 (15%)	330 (16%)	489 (14%)	0.250	207 (12%)	174 (12%)	162 (8%)	< 0.001
Chronic lung disease	1002 (43%)	918 (44%)	1403 (41%)	0.040	707 (43%)	680 (45%)	788 (41%)	0.042
Renal impairment	59 (3%)	64 (3%)	79 (2%)	0.222	33 (2%)	23 (2%)	13 (1%)	0.003
Atrial fibrillation	228 (10%)	183 (9%)	313 (9%)	0.403	689 (41%)	683 (46%)	1115 (58%)	< 0.001
Angina pectoris	898 (39%)	819 (39%)	1378 (40%)	0.638	375 (23%)	339 (23%)	416 (22%)	0.765
Recent myocardial infarction*	69 (3%)	54 (3%)	68 (2%)	0.045	37 (2%)	34 (2%)	25 (1%)	0.058
Congestive heart failure	548 (24%)	510 (24%)	749 (22%)	0.054	551 (33%)	519 (35%)	601 (31%)	0.129
Urgent or emergent surgery	59 (3%)	35 (2%)	25 (1%)	< 0.001	60 (4%)	43 (3%)	23 (1%)	< 0.001
Red blood cell transfusion, units^†^	2 (2–3)	2 (2–3)	3 (2–4)	< 0.001	3 (2–4)	2 (2–4)	2 (1–3)	< 0.001
Aortic valve diagnosis				< 0.001	NA			
Stenosis	1360 (59%)	1238 (59%)	1968 (57%)		NA			
Insufficiency	491 (21%)	455 (22%)	732 (21%)		NA			
Stenoinsufficiency	289 (12%)	287 (14%)	622 (18%)		NA			
Not specified	178 (8%)	122 (6%)	133 (4%)		NA			
Concurrent CABG	309 (13%)	353 (17%)	613 (18%)	< 0.001	NA			
Rheumatic mitral valve disease	NA				482 (29%)	528 (35%)	1033 (54%)	< 0.001
Concurrent atrial fibrillation surgery	NA				576 (35%)	674 (45%)	988 (51%)	< 0.001
Concurrent tricuspid valve repair	NA				463 (28%)	558 (37%)	1048 (55%)	< 0.001
Infective endocarditis	223 (10%)	187 (9%)	188 (5%)	< 0.001	26 (2%)	16 (1%)	24 (1%)	0.738

Values are presented as number (%), mean (SD), or median (interquartile range). *CABG* Coronary artery bypass grafting

*Diagnosed within three months before surgery

^†^During the hospitalisation for surgery

### Aortic valve replacement

A total of 7875 cases of AVR were performed in 94 centers with 2318, 2102, and 3455 patients undergoing AVR in 75 low-, 14 medium-, and 5 high-volume centers, respectively. The median (IQR) case volume was 6 (3–11), 33 (25–45), and 176 (116–184) in the low-, medium-, and high-volume centers, respectively (Table 1). Infective endocarditis was less frequent in the high-volume centers (188/3455, 5.4%) compared to the medium- (187/2102, 8.9%; *P* < 0.001) and low-volume centers (223/2318, 9.6%; *P* < 0.001).

**Fig. 1:**
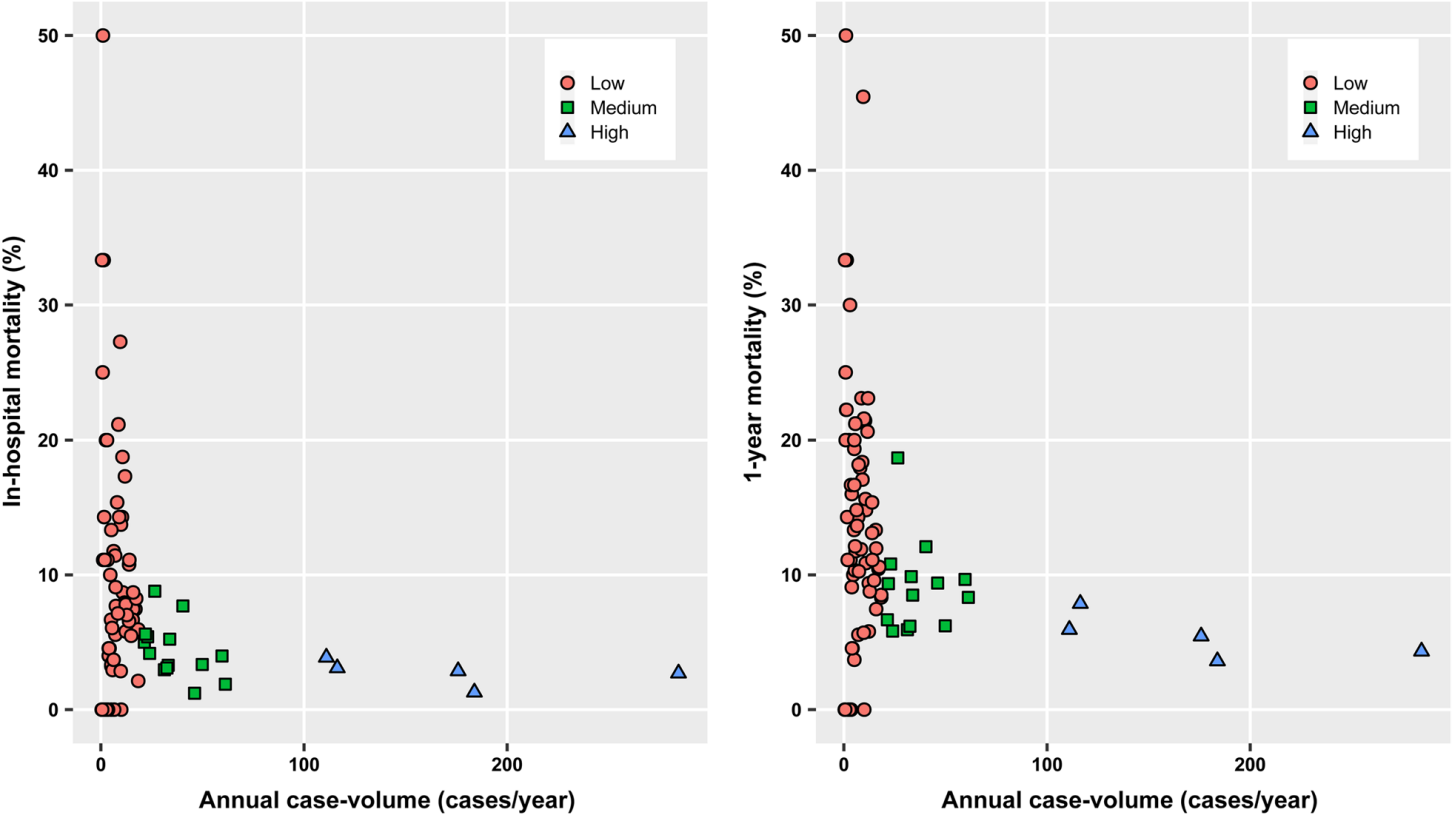
**a** In-hospital and **b** 1 year mortality rate after aortic valve replacement

In-hospital mortality rate of each AVR center is shown in Fig. 1a. When compared to the high-volume centers, the unadjusted odds ratios (ORs) [95% confidence interval (CI)] of the medium- and low-volume centers were 1.56 (1.15–2.11; *P* = 0.004) and 3.38 (2.61–4.36; *P* < 0.001), respectively (Additional file 1: Table S1). Multivariable logistic regression model showed that the adjusted risk of in-hospital mortality was significantly higher in the medium- (OR 1.53, 95% CI 1.09–2.15; *P* = 0.013) and the low-volume (OR 2.31, 95% CI 1.73–3.09; *P* < 0.001) centers compared to the high-volume centers (Table 2).

**Table 2:** Multivariable logistic regression analyses for in-hospital and 1 year mortality after aortic valve replacement

	In-hospital mortality			1 year mortality		
	OR	95% CI	*P*	OR	95% CI	*P*
Case volume strata						
High-volume (> 70 cases/year)	Reference			Reference		
Medium-volume (20–70 cases/year)	1.53	1.09–2.15	0.013	1.92	1.51–2.43	< 0.001
Low-volume (< 20 cases/year)	2.31	1.73–3.09	< 0.001	2.21	1.77–2.74	< 0.001
Age, years						
18–49	Reference			Reference		
50–59	1.52	0.75–3.07	0.246	1.43	0.86–2.40	0.172
60–69	1.58	0.81–3.08	0.182	1.66	1.02–2.70	0.041
70–79	3.04	1.57–5.87	< 0.001	3.03	1.88–4.88	< 0.001
≥ 80	4.05	2.00–8.18	< 0.001	4.42	2.65–7.38	< 0.001
Female	1.40	1.10–1.80	0.007	1.01	0.84–1.21	0.932
Hypertension	0.83	0.62–1.11	0.211	0.89	0.72–1.11	0.308
Dyslipidaemia	1.11	0.85–1.45	0.453	0.98	0.80–1.20	0.872
Diabetes mellitus	1.11	0.83–1.47	0.490	1.21	0.98–1.50	0.081
Extracardiac arteriopathy	1.13	0.83–1.53	0.447	1.07	0.85–1.35	0.576
Chronic lung disease	1.16	0.91–1.49	0.227	1.17	0.97–1.40	0.098
Renal impairment	2.96	1.91–4.59	< 0.001	2.90	2.00–4.20	< 0.001
Atrial fibrillation	1.55	1.07–2.23	0.020	1.41	1.07–1.86	0.016
Angina pectoris	0.98	0.76–1.27	0.880	1.03	0.85–1.25	0.742
Recent myocardial infarction*	1.32	0.73–2.42	0.362	1.06	0.65–1.75	0.815
Congestive heart failure	1.05	0.79–1.40	0.726	1.10	0.89–1.36	0.368
Urgent or emergent surgery	2.68	1.48–4.86	0.001	1.60	0.93–2.74	0.090
Red blood cell transfusion, units^†^						
0–1	Reference			Reference		
2–3	2.76	1.19–6.39	0.018	1.56	1.07–2.27	0.020
4–5	7.35	3.15–17.15	< 0.001	3.20	2.16–4.76	< 0.001
≥ 6	53.32	23.13–122.91	< 0.001	16.97	11.40–25.24	< 0.001
Aortic valve diagnosis						
Stenosis	Reference			Reference		
Insufficiency	0.99	0.68–1.44	0.939	0.88	0.67–1.16	0.363
Stenoinsufficiency	0.88	0.61–1.27	0.501	1.05	0.81–1.36	0.728
Not specified	1.61	1.04–2.50	0.033	1.34	0.94–1.91	0.106
Concurrent CABG	1.40	1.05–1.76	0.022	1.27	1.02–1.58	0.035
Infective endocarditis	2.29	1.53–3.43	< 0.001	2.36	1.71–3.25	< 0.001
Surgery year	0.99	0.94–1.04	0.648	1.00	0.96–1.04	0.948

*OR* Odds ratio; *CI* Confidence interval; *CABG* Coronary artery bypass grafting

*Diagnosed within three months before surgery

^†^During the hospitalisation for surgery

In the low-, medium-, and high-volume centers, 1 year mortality rates after AVR were 13.2% (306/2318), 9.0% (189/2102), and 4.9% (171/3455), respectively, with the overall rate of 8.5% (666/7875). One-year mortality rate of each AVR center is presented in Fig. 1b. After adjustment, 1 year mortality was still significantly higher in the medium- (OR 1.92, 95% CI 1.51–2.42, *P* < 0.001) and low-volume (OR 2.21, 95% CI 1.77–2.74, *P* < 0.001) centers compared to the high-volume centers (Table 2).

**Fig. 2:**
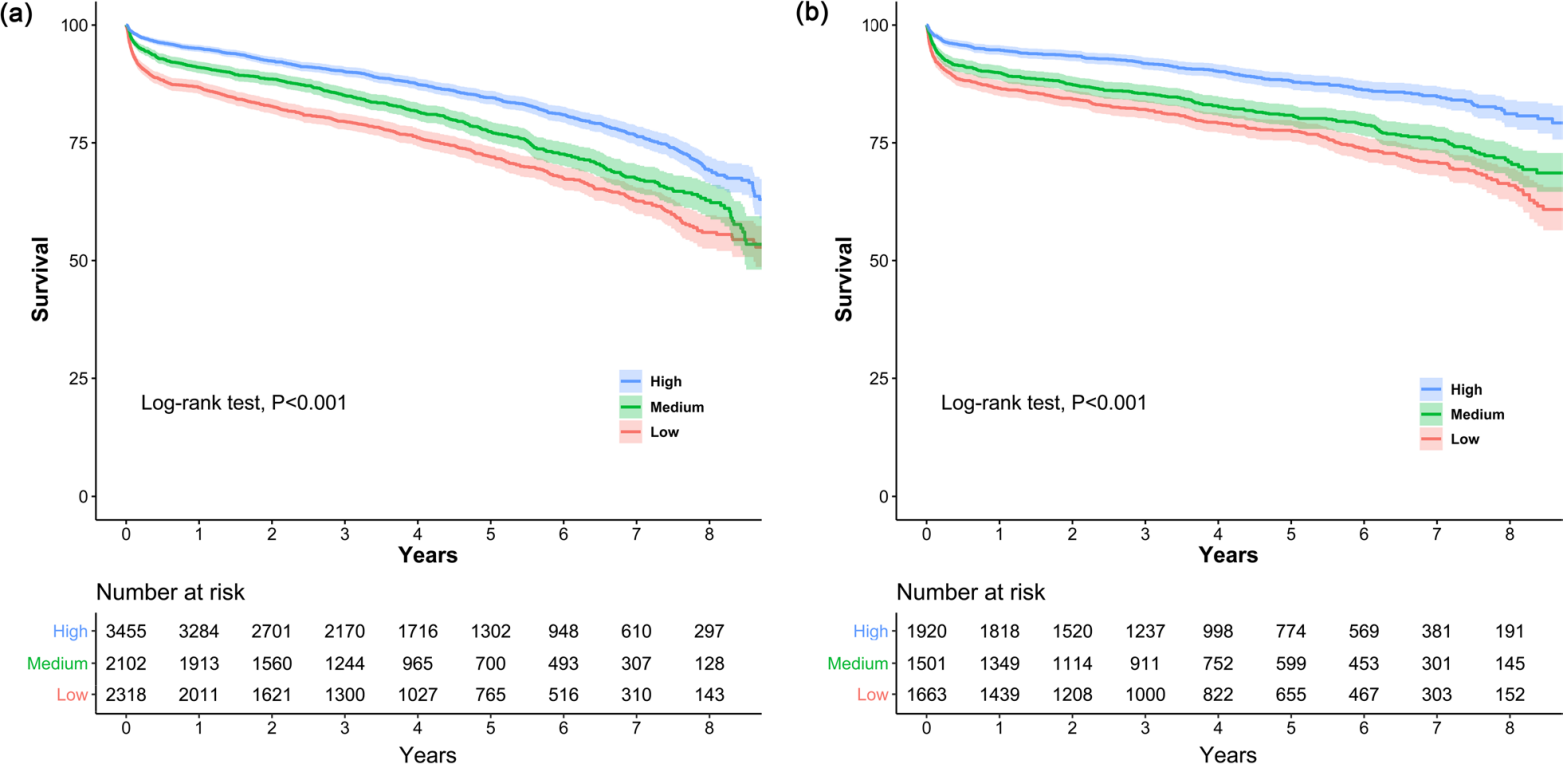
Kaplan–Meier survival curves for all-cause mortality after **a** aortic valve replacement and **b** mitral valve replacement

With a median (IQR) follow-up duration of 3.8 (2.0–6.0) years, survival rate after AVR was lower in the medium- and low-volume centers than in the high-volume centers (Fig. 2a , *P* < 0.001). The adjusted risk of cumulative all-cause mortality was also significantly higher in the medium- [hazard ratio (HR), 1.47; 95% CI, 1.28–1.68; *P* < 0.001] and low-volume centers (HR, 1.55; 95% CI, 1.388–1.74; *P* < 0.001) compared to the high-volume centers (Additional file 1: Table S2).

### Mitral valve replacement

In all, 5084 cases of MVR were conducted in 96 centers with 1663, 1501, and 1920 patients undergoing MVR in 76 low-, 15 medium-, and 5 high-volume centers, respectively. The median (IQR) case volumes were 4 (2–6), 18 (17–25), and 83 (67–115) in the low-, medium-, and high-volume centers, respectively (Table 1). Preoperative extracardiac arteriopathy was less common in the high-volume centers (162/1920, 8%) than in the medium- (174/1501, 12%) and low-volume centers (207/1663, 12%; *P* < 0.001). Rheumatic mitral valve pathology, concurrent atrial fibrillation surgery, and concurrent tricuspid valve repair were more frequent in the high-volume centers than the others.

**Fig. 3:**
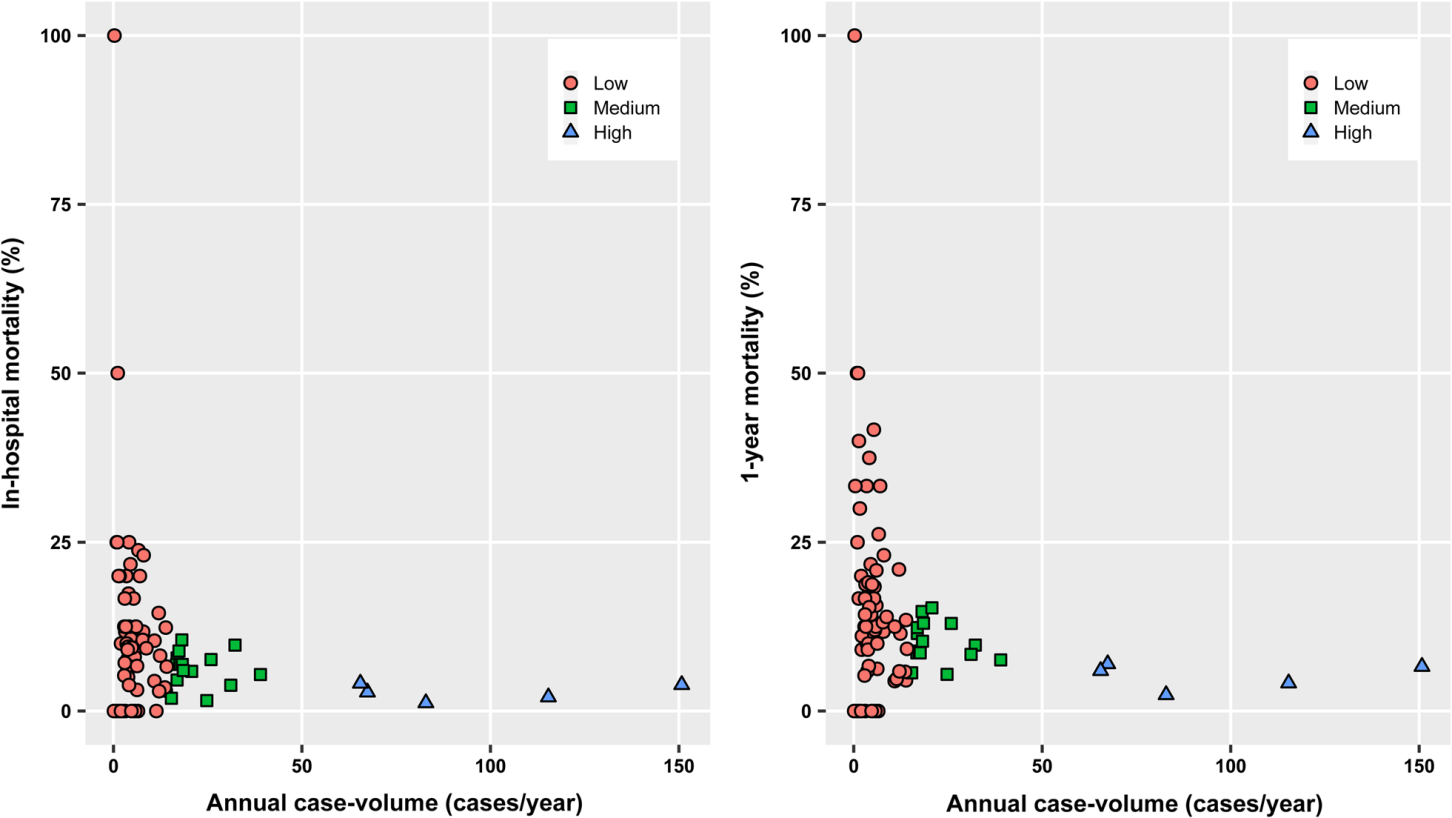
**a** In-hospital and **b** 1 year mortality rate after mitral valve replacement

In-hospital mortality rate of each MVR center is presented in Fig. 3a. When compared to the high-volume centers, the unadjusted ORs (95% CI) of the medium- and low-volume centers were 2.22 (1.59–3.12; *P* < 0.001) and 3.42 (2.50–4.68; *P* < 0.001), respectively (Additional file 1: Table S1). The adjusted ORs (95% CI) were 1.97 (1.35–2.88; *P* < 0.001) and 2.29 (1.60–3.27; *P* < 0.001), respectively (Table 3).

**Table 3:** Multivariable logistic regression analyses for in-hospital and 1 year mortality after mitral valve replacement

	In-hospital mortality			1 year mortality		
	OR	95% CI	*P*	OR	95% CI	*P*
Case volume strata						
High-volume (> 40 cases/year)	Reference			Reference		
Medium-volume (15–40 cases/year)	1.97	1.35–2.88	< 0.001	1.83	1.36–2.46	< 0.001
Low-volume (< 15 cases/year)	2.29	1.60–3.27	< 0.001	1.95	1.46–2.60	< 0.001
Age, years						
18–49	Reference			Reference		
50–59	1.51	0.91–2.49	0.111	1.54	1.03–2.32	0.037
60–69	1.63	1.01–2.66	0.047	1.97	1.33–2.91	0.001
70–79	3.48	2.16–5.60	< 0.001	3.65	2.46–5.40	< 0.001
≥ 80	7.77	4.12–14.64	< 0.001	9.40	5.52–16.01	< 0.001
Female	1.18	0.89–1.57	0.246	1.05	0.84–1.33	0.660
Hypertension	0.93	0.68–1.27	0.637	0.98	0.75–1.26	0.845
Dyslipidaemia	1.15	0.82–1.61	0.408	0.95	0.72–1.26	0.747
Diabetes mellitus	1.28	0.90–1.81	0.168	1.21	0.90–1.63	0.200
Extracardiac arteriopathy	1.18	0.81–1.71	0.387	1.47	1.09–1.99	0.013
Chronic lung disease	0.76	0.58–1.00	0.053	0.88	0.70–1.09	0.241
Renal impairment	5.27	2.89–9.60	< 0.001	6.79	3.74–12.33	< 0.001
Angina pectoris	0.97	0.71–1.34	0.859	1.02	0.78–1.33	0.895
Recent myocardial infarction*	1.18	0.54–2.58	0.677	1.09	0.55–2.14	0.808
Congestive heart failure	1.07	0.79–1.45	0.654	1.12	0.88–1.43	0.369
Urgent or emergent surgery	1.64	0.95–2.84	0.078	1.69	1.03–2.75	0.036
Red blood cell transfusion, units^†^						
0–1	Reference			Reference		
2–3	4.82	1.50–15.47	0.008	1.36	0.84–2.19	0.215
4–5	14.48	4.51–46.49	< 0.001	3.45	2.11–5.62	< 0.001
≥ 6	74.06	23.22–236.14	< 0.001	16.92	10.39–27.55	< 0.001
Rheumatic mitral valve disease	0.81	0.59–1.12	0.199	0.85	0.66–1.09	0.204
Concurrent atrial fibrillation surgery	0.85	0.62–1.15	0.267	0.76	0.59–0.97	0.029
Concurrent tricuspid valve repair	0.98	0.73–1.31	0.890	0.99	0.78–1.26	0.932
Infective endocarditis	0.46	0.13–1.71	0.248	0.24	0.06–0.87	0.030
Surgery year	0.99	0.93–1.05	0.648	0.96	0.92–1.01	0.840

*OR* Odds ratio; *CI* Confidence interval

*Diagnosed within three months before surgery

^†^During the hospitalisation for surgery

While the overall 1 year mortality rates following MVR was 9.4% (476/5084), 1 year mortality rates of the low-, medium-, and high-volume centers were 13.4% (223/1663), 10.1% (152/1501), and 5.3% (101/1920), respectively. One-year mortality rates of individual centers are shown in Fig. 3b. On the multivariable analysis, the risk of 1 year mortality was significantly greater in the medium- (OR 1.83, 95% CI 1.36–2.46, *P* < 0.001) and low-volume centers (OR 1.95, 95% CI 1.46–2.60, *P* < 0.001) compared to the high-volume centers (Table 3).

The Kaplan–Meier curves for cumulative all-cause mortality are presented in Fig. 2b. The median (IQR) duration of follow-up was 4.1 (2.0–6.5) years. Compared to the high-volume centers, survival rate was lower in the medium- and low-volume centers (*P* < 0.001). The adjusted HRs (95% CI) of the medium- and low-volume centers were 1.54 (1.30–1.84; *P* < 0.001) and 1.58 (1.34–1.88; *P* < 0.001), respectively, when referenced to the high-volume centers (Additional file 1: Table S2).

## Discussion

In this nationwide observational study, institutional case volume was found to be independently associated with postoperative mortality in patients undergoing AVR and MVR. The risk of in-hospital death after AVR and MVR was significantly greater in the low- and medium-volume centers compared to the high-volume centers. Similar association was found between institutional case volume and the risk of 1 year mortality following AVR and MVR. The risks of cumulative all-cause mortality after AVR and MVR with the median follow-up of 4 years were also significantly higher in the low- and medium-volume centers compared to the high-volume centers.

Cardiac surgery is a complex, high-risk procedure that requires comprehensive medical services based on surgeon and center’s capability to optimize patient outcomes. Despite noticeable development during the past decades, mortality after all types of cardiac surgery is still high compared to non-cardiac surgery, surpassing 6% [15]. In the US alone, nearly 300,000 cases of cardiac surgery are performed each year, imposing a burden in regard to medical expenses to both society and individuals [15]. Therefore, efficient distribution and utilization of medical resources for cardiac surgery are of utmost concern in many countries. Concentrating the limited resources to a few dedicated centers or healthcare providers, regionalization of cardiac surgery may be an effective approach to this end [18]. However, regionalization inevitably sacrifices access to care to some extent for patients living far away, thus necessitating in-depth academic or socioeconomic discussions [19, 20]. Establishing data on the volume-outcome relationship may therefore be a starting point for these debates in the field of cardiac surgery.

While the components and their interactions that drive the volume-outcome relationship remain unclear, the association has been consistently shown in various non-cardiac surgeries including carotid endarterectomy [6], lung resection [4, 21], esophagectomy [2, 21], and major abdominal surgeries [2, 4, 22, 23]. The association has been evaluated for cardiac surgery as well, but most studies were performed in patients undergoing coronary artery bypass grafting, yielding conflicting results [7, 8, 24–26]. Evidences of the volume-outcome relationship can be one of the references when determining concentration of high-cost, high-risk surgical procedures, such as cardiac surgery, to a few centers with adequate operative outcomes. At the same time, a significant volume-outcome relationship per se may justify such administrative policy. Indeed, although not compulsory, the European Association for Cardio-Thoracic Surgery noted in their clinical statement that a cardiac surgery center should perform a minimum of 500 cardiac surgical procedures in order to maintain satisfactory patient outcomes and that smaller-volume centers should have robust governance [27]. Likewise, the American College of Surgeons recommended that an institutional case volume for cardiac surgeries requiring cardiopulmonary bypass be at least 100–125 per year [28].

The present study found a volume-outcome relationship where the risks of postoperative mortality after AVR and MVR were significantly greater in lower-volume centers than in higher-volume centers in Korea. However, the authors do not intend to urge regionalization of cardiac surgery with the results of this study. Previous studies also reported a significant relationship between institutional case volume and mortality after AVR [3, 10, 11, 29] or MVR [3, 29]. However, the differences in operative mortality (postoperative death during hospital stay or within 30 days) after AVR and MVR between centers with high- (the highest quintile) and low-volume (the lowest quintile) have been reported to be modest at best [29]. Operative mortality in high-risk patients was similar between high- and low-volume centers (AVR, 12.2 vs. 16.0%; MVR, 19.9% vs. 24.9%) [29]. Moreover, hospital length of stay was even longer in higher volume centers and 30-day re-admission rates were not affected by case volume [30]. Similarly, in-hospital mortality was not different significantly between case volume strata [7.2% in low-volume centers (≤ 60 cases/year) and 5.1% in high-volume centers (> 180 cases/year)], although logistic regression analysis showed that case volume was associated with the risk of in-hospital mortality [10]. The discrepancy between these studies and our study may have stemmed from wide range of mortality in the low-volume centers in our study. As seen in Figs. 1 and 3, not all low-volume centers showed a high mortality. A significant number of low-volume centers showed clinical outcomes comparable to higher-volume centers, which is already well known [31–33]. Surgical procedure and perioperative care practiced by these centers may be a guide to other low volume centers with suboptimal patient outcome.

There are several limitations to consider in the present study. First, the results of the retrospective study may have indicated merely an association between institutional case volume and surgical outcomes rather than a cause-effect relationship. Although various potential confounders were adjusted for in the analysis, a bias still may be in play. The bias, however, may have been attenuated fairly because all cases of AVR and MVR conducted in Korea during the study period were included in this study. Second, several important clinical data, such as laboratory findings, perioperative hemodynamic profile, type of prosthetic valve, and postoperative anticoagulation, were not adjusted for because the NHIS database lacked these data. The Society of Thoracic Surgeons score and European System for Cardiac Operative Risk Evaluation II were also not available although most of the variables constituting both scores systems were adjusted for in this study. Third, the determination of cut-off values for the case volume strata relied only on the visual inspection of the scatterplot (Fig. 1), which was somewhat arbitrary. Currently, there are no regulations requiring a minimum procedure volume for cardiac surgery centers in Korea. To provide supportive data on the minimum requirement, future studies with more granular clinical data are required. Fourth, case volume of individual cardiac surgeons was not analyzed. Although highly systemized management with multidisciplinary approach is crucial, operative outcomes of high-risk surgeries still may heavily depend on individual surgeon’s experience. A previous study suggested that the observed association between institutional case volume and outcomes were mediated largely by surgeon case volume [6]. However, many cardiac surgery centers in Korea, including high volume centers, generally have only a few cardiac surgeons. It is difficult to assess the impact of individual surgeon in our study as relevant data was lacking in the database. The high variability in outcomes in low volume centers may be explained partially by individual variation in surgical skill. Future studies seeking the independent effect of surgeon volume are required.

## Conclusions

In conclusion, a lower institutional case volume is significantly associated with increased risk of postoperative mortality including in-hospital, 1 year, and cumulative all-cause death in patients undergoing AVR and MVR. The results of this study indicate that discussion on regionalization of cardiac surgery may be considered to optimize clinical outcomes.

## Supplementary Information

**Additional file 1: Table S1.** Univariable logistic regression analyses for in-hospital mortality. **Table S2.** Multivariable Cox regression analyses for cumulative postoperative all-cause mortality.

## Data Availability

The data that support the findings of this study are available from the National Health Insurance Service of Korea but restrictions apply to the availability of these Korean administrative data, which were used under license for the current study, and so are not publicly available.

## Abbreviations

AVR: Aortic valve replacement
CI: Confidence interval
IQR: Interquartile range
MVR: Mitral valve replacement
NHIS: National health insurance service
OR: Odds ratio
