# Supplementary material for: Institutional case volume and mortality after aortic and mitral valve replacement: a nationwide study in two Korean cohorts
Source: J Cardiothorac Surg. 2022 Aug 20;17:190. doi: 10.1186/s13019-022-01945-0 (PMC9392916; doi:10.1186/s13019-022-01945-0)
Supplement: Supplementary file 1 — Additional file 1: Table S1. Univariable logistic regression analyses for in-hospital mortality. Table S2. Multivariable Cox regression analyses for cumulative postoperative all-cause mortality. [file 13019_2022_1945_MOESM1_ESM.docx]

**Supplementary Table 1.** Univariable logistic regression analyses for in-hospital mortality

|  | Aortic valve replacement | | |  | Mitral valve replacement | | |
| --- | --- | --- | --- | --- | --- | --- | --- |
|  | OR | 95% CI | P |  | OR | 95% CI | P |
| Case volume strata |  |  |  |  |  |  |  |
| High-volume^a^ | Reference | | |  | Reference | | |
| Medium-volume^b^ | 1.56 | 1.15–2.11 | 0.004 |  | 2.22 | 1.59–3.12 | <0.001 |
| Low-volume^c^ | 3.38 | 2.61–4.36 | <0.001 |  | 3.42 | 2.50–4.68 | <0.001 |
| Age, years |  |  |  |  |  |  |  |
| 18–49 | Reference | | |  | Reference | | |
| 50–59 | 1.55 | 0.83–2.89 | 0.174 |  | 1.24 | 0.79–1.94 | 0.350 |
| 60–69 | 1.47 | 0.82–2.65 | 0.195 |  | 1.69 | 1.11–2.57 | 0.015 |
| 70–79 | 3.10 | 1.79–5.38 | <0.001 |  | 4.18 | 2.81–6.20 | <0.001 |
| ≥80 | 4.66 | 2.61–8.34 | <0.001 |  | 9.58 | 5.70–16.12 | <0.001 |
| Female | 1.57 | 1.27–1.93 | <0.001 |  | 1.09 | 0.89–1.38 | 0.482 |
| Hypertension | 1.38 | 1.09–1.75 | 0.007 |  | 1.33 | 1.04–1.70 | 0.022 |
| Dyslipidemia | 1.37 | 1.11–1.70 | 0.004 |  | 1.50 | 1.14–1.96 | 0.003 |
| Diabetes | 1.56 | 1.23–1.98 | <0.001 |  | 2.24 | 1.69–2.97 | <0.001 |
| Extracardiac arteriopathy | 1.62 | 1.25–2.09 | <0.001 |  | 1.79 | 1.31–2.45 | <0.001 |
| Chronic lung disease | 1.36 | 1.10–1.68 | 0.004 |  | 0.99 | 0.79–1.26 | 0.954 |
| Renal impairment | 6.15 | 4.32–8.78 | <0.001 |  | 13.26 | 8.11–21.67 | <0.001 |
| Atrial fibrillation | 1.68 | 1.24–2.28 | <0.001 |  | NA | | |
| Angina pectoris | 1.18 | 0.96–1.46 | 0.121 |  | 1.43 | 1.10–1.85 | 0.007 |
| Recent myocardial infarction^d^ | 2.48 | 1.54–3.99 | <0.001 |  | 1.85 | 0.95–3.60 | 0.070 |
| Previous PCI | 3.37 | 2.00–5.68 | <0.001 |  | NA | | |
| Congestive heart failure | 1.32 | 1.04–1.66 | 0.022 |  | 1.09 | 0.86–1.40 | 0.470 |
| Urgent or emergent surgery | 4.60 | 2.84–7.47 | <0.001 |  | 3.49 | 2.17–5.62 | <0.001 |
| Infective endocarditis | 3.00 | 2.28–3.97 | <0.001 |  | 0.74 | 0.23–2.38 | 0.618 |
| RBC transfusion, units^e^ |  |  |  |  |  |  |  |
| 0–1 | Reference | | |  | Reference | | |
| 2–3 | 3.97 | 1.72–9.16 | 0.001 |  | 6.93 | 2.17–22.13 | 0.001 |
| 4–5 | 14.45 | 6.27–33.27 | <0.001 |  | 25.33 | 7.97–80.57 | <0.001 |
| ≥6 | 124.70 | 54.99–272.79 | <0.001 |  | 144.06 | 45.72–453.95 | <0.001 |
| Aortic valve diagnosis |  |  |  |  |  | | |
| Stenosis | Reference | | |  | NA | | |
| Insufficiency | 0.77 | 0.57–1.03 | 0.073 |  | NA | | |
| Steno-insufficiency | 0.81 | 0.59–1.12 | 0.209 |  | NA | | |
| Not specified | 2.36 | 1.68–3.31 | <0.001 |  | NA | | |
| Concurrent CABG | 2.15 | 1.70–2.72 | <0.001 |  | NA | | |
| Rheumatic mitral valve disease | NA | | |  | 0.43 | 0.33–0.57 | <0.001 |
| Concurrent atrial fibrillation surgery | NA | | |  | 0.49 | 0.38–0.63 | <0.001 |
| Concurrent tricuspid valve repair | NA | | |  | 0.89 | 0.70–1.13 | 0.329 |
| Surgery year | 1.01 | 0.96–1.06 | 0.684 |  | 1.00 | 0.96–1.06 | 0.845 |

OR, odds ratio; CI, confidence interval; PCI, percutaneous coronary intervention; CABG, coronary artery bypass grafting.

^a^Centers with ≥70 and ≥40 cases/year of aortic valve and mitral valve replacement, respectively.

^b^Centers with 20­–70 and 15–40 cases/year of aortic valve and mitral valve replacement, respectively.

^c^Centers with <20 and <15 cases/year of aortic valve and mitral valve replacement, respectively.

^d^Diagnosed within 3 months before surgery.

^e^During the hospitalization for surgery.

**Supplementary Table 2.** Multivariable Cox regression analyses for cumulative postoperative all-cause mortality

|  | Aortic valve replacement | | |  | Mitral valve replacement | | |
| --- | --- | --- | --- | --- | --- | --- | --- |
|  | HR | 95% CI | P |  | HR | 95% CI | P |
| Case volume strata |  |  |  |  |  |  |  |
| High-volume^a^ | Reference | | |  | Reference | | |
| Medium-volume^b^ | 1.47 | 1.28–1.68 | <0.001 |  | 1.54 | 1.30–1.84 | <0.001 |
| Low-volume^c^ | 1.55 | 1.38–1.74 | <0.001 |  | 1.58 | 1.34–1.88 | <0.001 |
| Age, years |  |  |  |  |  |  |  |
| 18–49 | Reference | | |  | Reference | | |
| 50–59 | 1.57 | 1.15–2.16 | 0.005 |  | 1.62 | 1.24–2.12 | <0.001 |
| 60–69 | 2.32 | 1.74–3.12 | <0.001 |  | 2.47 | 1.92–3.18 | <0.001 |
| 70–79 | 4.12 | 3.09–5.50 | <0.001 |  | 4.64 | 3.61–5.96 | <0.001 |
| ≥80 | 7.10 | 5.25–9.62 | <0.001 |  | 8.88 | 6.41–12.30 | <0.001 |
| Female | 0.78 | 0.70–0.86 | <0.001 |  | 0.84 | 0.73–0.96 | 0.010 |
| Hypertension | 1.02 | 0.91–1.15 | 0.700 |  | 0.97 | 0.83–1.13 | 0.701 |
| Dyslipidemia | 0.99 | 0.89–1.10 | 0.782 |  | 0.96 | 0.81–1.14 | 0.660 |
| Diabetes mellitus | 1.33 | 1.19–1.48 | <0.001 |  | 1.55 | 1.32–1.83 | <0.001 |
| Extracardiac arteriopathy | 1.17 | 1.04–1.33 | 0.008 |  | 1.13 | 0.94–1.35 | 0.193 |
| Chronic lung disease | 1.24 | 1.12–1.36 | <0.001 |  | 0.91 | 0.80–1.04 | 0.159 |
| Renal impairment | 3.63 | 3.00–4.39 | <0.001 |  | 3.51 | 2.56–4.81 | <0.001 |
| Atrial fibrillation | 1.33 | 1.14–1.54 | <0.001 |  | NA | | |
| Angina pectoris | 0.98 | 0.88–1.08 | 0.767 |  | 1.07 | 0.92–1.24 | 0.397 |
| Recent myocardial infarction^d^ | 1.19 | 0.92–1.53 | 0.626 |  | 1.00 | 0.68–1.46 | 0.986 |
| Congestive heart failure | 1.06 | 0.95–1.19 | 0.308 |  | 1.25 | 1.08–1.44 | 0.002 |
| Urgent or emergent surgery | 1.39 | 0.99–1.94 | 0.309 |  | 1.50 | 1.11–2.03 | 0.008 |
| Red blood cell transfusion, units^e^ |  |  |  |  |  |  |  |
| 0–1 | Reference | | |  | Reference | | |
| 2–3 | 1.29 | 1.09–1.54 | 0.005 |  | 1.25 | 0.97–1.62 | 0.083 |
| 4–5 | 1.88 | 1.56–2.27 | <0.001 |  | 1.99 | 1.52–2.61 | <0.001 |
| ≥6 | 5.11 | 4.19–6.23 | <0.001 |  | 5.39 | 4.10–7.08 | <0.001 |
| Aortic valve diagnosis |  |  |  |  |  | | |
| Stenosis | Reference | | |  | NA | | |
| Insufficiency | 0.92 | 0.79–1.06 | 0.237 |  | NA | | |
| Stenoinsufficiency | 0.91 | 0.79–1.05 | 0.211 |  | NA | | |
| Not specified | 1.12 | 0.91–1.38 | 0.220 |  | NA | | |
| Concurrent CABG | 1.19 | 1.06–1.34 | 0.003 |  | NA | | |
| Rheumatic mitral valve disease | NA | | |  | 0.90 | 0.77–1.05 | 0.161 |
| Concurrent atrial fibrillation surgery | NA | | |  | 0.72 | 0.62–0.84 | <0.001 |
| Concurrent tricuspid valve repair | NA | | |  | 1.10 | 0.95–1.27 | 0.207 |
| Infective endocarditis | 1.65 | 1.36–2.00 | <0.001 |  | 0.63 | 0.34–1.18 | 0.151 |

HR, hazard ratio; CI, confidence interval; CABG, coronary artery bypass grafting.

^a^Centers with >70 and >40 cases/year of aortic valve and mitral valve replacement, respectively.

^b^Centers with 20­–70 and 15–40 cases/year of aortic valve and mitral valve replacement, respectively.

^c^Centers with <20 and <15 cases/year of aortic valve and mitral valve replacement, respectively.

^d^Diagnosed within 3 months before surgery.

^e^During the hospitalization for surgery.
